# Insights into early ontogenesis: characterization of stress and development key genes of pikeperch (*Sander lucioperca*) in vivo and in vitro

**DOI:** 10.1007/s10695-021-00929-6

**Published:** 2021-02-09

**Authors:** Nadine Schäfer, Yagmur Kaya, Henrike Rebl, Marcus Stüeken, Alexander Rebl, Julien A. Nguinkal, George P. Franz, Ronald M. Brunner, Tom Goldammer, Bianka Grunow, Marieke Verleih

**Affiliations:** 1 Institute of Genome Biology, Leibniz Institute for Farm Animal Biology (FBN), Wilhelm-Stahl-Allee 2, 18196 Dummerstorf, Germany; 2 Institute of Muscle Biology and Growth, Leibniz Institute for Farm Animal Biology (FBN), Wilhelm-Stahl-Allee 2, 18196 Dummerstorf, Germany; 3 Department of Cell Biology, Rostock University Medical Center, 18059 Rostock, Germany; 4 Institute of Fisheries, Department of Aquaculture, Mecklenburg-Vorpommern Research Centre for Agriculture and Fisheries, 17194 Hohen Wangelin, Germany; 5 Faculty of Agriculture and Environmental Sciences, University of Rostock, 18059 Rostock, Germany

**Keywords:** Aquaculture, Animal welfare, Fish cell line, Early ontogenesis, Pikeperch, Stress response

## Abstract

There are still numerous difficulties in the successful farming of pikeperch in the anthropogenic environment of various aquaculture systems, especially during early developmental steps in the hatchery. To investigate the physiological processes involved on the molecular level, we determined the basal expression patterns of 21 genes involved in stress and immune responses and early ontogenesis of pikeperch between 0 and 175 days post hatch (dph). Their transcription patterns most likely reflect the challenges of growth and feed conversion. The gene coding for apolipoprotein A (*APOE*) was strongly expressed at 0 dph, indicating its importance for yolk sac utilization. Genes encoding bone morphogenetic proteins 4 and 7 (*BMP4*, *BMP7*), creatine kinase M (*CKM*), and SRY-box transcription factor 9 (*SOX9*) were highly abundant during the peak phases of morphological changes and acclimatization processes at 4–18 dph. The high expression of genes coding for peroxisome proliferator-activated receptors alpha and delta (*PPARA*, *PPARD*) at 121 and 175 dph, respectively, suggests their importance during this strong growth phase of juvenile stages. As an alternative experimental model to replace further in vivo investigations of ontogenetically important processes, we initiated the first approach towards a long-lasting primary cell culture from whole pikeperch embryos. The present study provides a set of possible biomarkers to support the monitoring of pikeperch farming and provides a first basis for the establishment of a suitable cell model of this emerging aquaculture species.

## Introduction

Pikeperch (*Sander lucioperca* L., 1758) is an important food fish in Europe. Due to its exceptionally soft flesh, rapid growth, and positive market acceptance, pikeperch is traded as an outstandingly high-quality fish. Consequently, it became an increasingly attractive freshwater species for European aquaculture (FAO 2018). For more than two decades, efforts have been made to improve the intensive production of pikeperch in recirculating aquaculture systems (RAS) (Hilge and Steffens 1996; Steenfeldt 2015; Policar et al. 2016). Nevertheless, pikeperch farming and especially hatcheries are hampered by difficulties during early ontogenesis, which comprises the central developmental stage of organogenesis, including myo-, skeleto-, and neurogenesis, as well as the phase of growth and the development of the immune system (Zapata et al. 2006; Alix et al. 2015). The embryonic phase of pikeperch ends with hatching at approximately 4 to 6 days post fertilization (dpf). It spans from shortly after the mouth opens and the mixotrophic feeding phase begins until the complete resolution of the yolk sac up to 7 dph. This is followed by the gradual adaptation to pelleted feed and transition to the juvenile stage (Ott et al. 2012a; Güralp et al. 2017). The three main bottlenecks of pikeperch farming are the conversion from endogenous to exogenous feed, the inflation of the swim bladder, and cannibalism (summarized in Steenfeldt 2015). This results in general problems such as animal malformations, impaired growth, and high mortality rates (Kestemont et al. 2007; Szkudlarek and Zakȩś 2007; Policar et al. 2016; Schaefer et al. 2017; Baekelandt et al. 2018; Schaefer et al. 2018).

Therefore, basic knowledge of the physiological processes that occur during pikeperch rearing is of major importance for improving aquaculture production. This includes the profiling of the basal gene expression pattern during the early developmental stages. Although pikeperch is considered to be highly stress susceptible (Németh et al. 2013; Baekelandt et al. 2018), the stress physiology of the species has received little attention thus far (Milla et al. 2015; Swirplies et al. 2019; Wang et al. 2019)^,^ especially in the early phases of development. This also applies to the immune response, which is well investigated in percid fishes such as yellow perch (*Perca flavescens*) or Eurasian perch (*Perca fluviatilis*) but is poorly documented in pikeperch. Current studies mainly focus on humoral stress and immune markers under certain husbandry conditions (Baekelandt et al. 2019; Baekelandt et al. 2020; Żarski et al. 2020), but alterations within gene expression during physiological changes of early ontogenesis have not been reported so far. In the present study, we investigated the transcription patterns of selected genes involved in stress and immune responses as well as the growth phase and the previously mentioned stages of early development. During organogenesis, the retinoid X receptor alpha (RXRA) is important for the development of the posterior brain, neural crest, and tail bud (He et al. 2009). Bone morphogenetic proteins such as BMP4 and BMP7 have been shown to be involved in skeletogenesis and the development of the immune system (Zapata et al. 2006). The growth phase is represented among others by the growth hormone receptor (GHR) (Calduch-Giner et al. 2003). Since pikeperch is particularly sensitive during the phase of feed conversion, genes important for lipid metabolism and the mobilization of energy reserves such as *APOE* (Otis et al. 2015) have been included. Common markers for stress such as the glucocorticoid receptor (NR3C1) have further been applied regarding the high impact of stressful events on the welfare status of pikeperch in early stages.

In addition to the in vivo studies, it is also our concern to advance the in vitro studies in fish research, as the efforts of the 3Rs (replacement, reduction, and refinement) have so far been highly limited in fish research. This statement is underlined by high numbers of experimental animals (2017: ~ 1,220,000 fish within the European Union [European Commission 2020]), which suggests that the establishment of alternative experimental models such as cell culture systems of the respective fish species are necessary. These 3R cell models can be used as an essential tool for detailed research purposes, such as for studying ontogenetically relevant processes or the effects of stress under controlled exogenous conditions. There are several commercially available cell lines from different teleost fishes (ATCC and ExPASy databases, reviewed by Lakra et al. 2011), including the cell lines established from walleye (*Sander vitreus*): WF1 (dermal sarcoma; BS TCL 65) and WF2 (whole fry (Wilensky, C and Bowser 2005); BS Cl 88) (IZSLER Brescia, Italy). A cell line from *S. lucioperca* is currently not available.

Therefore, the main goals of this study were (i) to characterize and evaluate key genes for development as well as stress response in the early ontogenesis of pikeperch, (ii) to initiate the first approach to generating a cell model from pikeperch derived out of whole embryos, and (iii) to analyze the suitability of an in vitro model for studying developmental processes in pikeperch.

## Materials and methods

### Fish sample material

Pikeperch (strain “Sachsen,” Germany) were bred and reared in RAS at the State Research Centre for Agriculture and Fisheries Mecklenburg-Vorpommern (Hohen Wangelin, Germany) within their normal production cycle from June 2018 until their transfer as fingerlings (108 dph) to the Experimental Animal Facility Aquaculture of the Leibniz Institute for Farm Animal Biology (FBN, Dummerstorf, Germany).

Seven matings of pikeperch were generated with a sex ratio of 2:1 or 1:1. The progeny was mixed and reared in a RAS system with a total volume of 9 m^3^. A pump with a capacity of 9 m^3^/h supplies eight fish tanks with a volume of 0.5 m^3^ each with a water exchange rate of 20%/h/tank. The system also consists of a drum filter (72-qm gauze), a heater, an electrically controlled water supply, a moving bed biofilter (50% of the total volume, granulate surface of 850 m^2^/m^3^), and pressure sensors for pump operation. The initial stocking density was 100 larvae/l. The water quality was ensured by continuous purification, UV disinfection, and daily monitoring of temperature, oxygen saturation, and pH value. Concentrations of NH^4+^, NO^2^, and NO^3−^ were determined twice per week in circulating systems of larvae and fingerlings. The photoperiod during hatchery was set at 24L:0D until day 45 and subsequently at 17L:7D (+ 1.5 h dusk and dawn). The feed included a copepod mix from 5 dph (Aquacopa, Germany), followed by *Artemia* nauplii from 7 dph, and enriched *Artemia* from 9 dph (both Inve, Belgium). From 15 dph onwards, dry feed was added until the complete conversion to dry feed (Otohime B1, PTAqua, Ireland) had taken place. Fish were transported to the Leibniz Institute for Farm Animal Biology in a transport box with an additional oxygen provision and in small groups for animal welfare reasons. The transport was followed by an acclimation period of at least two weeks to the local RAS system before sampling.

Eyed eggs were kept at ~ 15.5 °C, ~ 12.9 mg/l dissolved oxygen (DO), and a pH of ~ 8.0. To ensure clean water, approximately 20–30% of the water was renewed every day. Larvae were kept at ~ 15.7 °C, ~ 9.2 mg/l DO, ~ 0.1 mg/l NH^4+^, ~ 15.7 mg/l NO^3−^, ~ 0.2 mg/l NO^2−^, and a pH of ~ 8.7. Fingerlings were kept at ~ 21.1 °C, ~ 8.1 mg/l DO, < 0.001 mg/l NH^4+^, ~ 38.8 mg/l NO^3−^, ~ 0.07 mg/l NO^2−^, and a pH of ~ 8.5. For gene expression analysis, we sampled eyed eggs (0 dph/78 degree days (DD); three pools of *n* = 20), yolk sack larvae (4 dph/137 DD; three pools of *n* = 20), larvae fed with *Artemia* spp*.* (7 dph/252 DD; three pools of *n* = 30), larvae fed with dry feed (18 dph/481 DD; three pools of *n* = 30), and liver tissues from 121- to 175-dph-old fingerlings (each *n* = 3 individuals). Prior to tissue sampling, we anesthetized fingerlings with 2-phenoxyethanol (50 mg/l). According to the recommendations of the German Animal Welfare Act (§ 4(3) TierSchG), fishes were then stunned by a blow on the head and killed directly by a bleed cut in the heart as well as cutting of the spinal cord posterior to the head. Collected material was snap-frozen in liquid nitrogen and stored at − 80 °C until further investigation.

### Cell isolation

By generating an embryonic cell line, we aimed to create an alternative to the use of embryos for experimental purposes. Nine days after fertilization and at an age of 125 DD, 12 embryos from pikeperch*,* with a length of about 4.7 ± 0.07 mm, were used for cell isolation. At 125 DD, the embryos were at eyed egg stage (= 0 dph) which was confirmed by observing them under the microscope. Based on the trypsinization technique, which we had already applied for the Atlantic sturgeon (*Acipenser oxyrinchus*) cell line AOXlar7 (Grunow et al. 2011a), we isolated the specimen from the eggshell and decapitated it using forceps. After washing three times with 1× DPBS (Dulbecco’s Phosphate-Buffered Saline; PAN-Biotech), we transferred whole embryos into a 1.5-ml tube and dissociated each with scissors and 100 μl of 0.1% trypsin/EDTA solution (Gibco Life Technologies) for one to two minutes. Digestion was terminated by adding triple the volume of Dulbecco’s modified Eagle medium (DMEM, with 4.5 g/l glucose and l-glutamine; Lonza BioWhittaker) supplemented with 20% FBS (fetal bovine serum, PAN-Biotech) and a 1% (v/v) penicillin/streptomycin solution (Gibco). After centrifugation for 5 min at 130*g*, cells were resuspended in cell culture medium supplemented with additional antibiotics (Gentamycin: 0.1 mg/ml and Kanamycin: 0.1 mg/ml; Biochrom AG). Additionally, an antimycotic agent (Amphotericin: 250 μg/ml, Biochrom AG) was added. Cells were placed into 6-well culture plates (TPP) and incubated at 20 °C and 2.5% CO_2_. In the following, this long-term cell culture is called SLUlar1.

### Cell culture

For SLUlar1 cells, the medium was exchanged every 2 days for the first 2 weeks. Afterwards, half the medium was exchanged once or twice per week, without the use of additional antibiotics. Cells were sub-cultured at a ratio of 1:2, when confluence of 80 to 90% was reached. Therefore, the cells were washed with DPBS and incubated with 0.1% trypsin/EDTA solution at 37 °C for 1 to 2 min. Trypsinization was stopped by adding double the volume of the cell culture medium. Cells were centrifuged for 5 min at 130 g, and the cell pellet was resuspended in a new culture medium and transferred into new culture dishes. From passage two onwards, T25 flasks (TPP) were applied. Cell attachment and cell morphology were visualized under the inverted phase-contrast microscope (Motic AE2000), and pictures were taken with Motic Images Plus 3.0 Software. Images were optimized with Adobe Photoshop CS 4 (Adobe Inc.).

We tested the freezing and thawing of SLUlar1 cells as follows: After trypsinization, cells were resuspended in precooled (+ 4 °C) freezing medium (9:1 ratio of precooled FBS:DMSO [Dimethylsulfoxid; Roth]), placed into 1.2-ml cryogenic vials (Roth; ~ 7.5 × 10^5^ ± 0.4 × 10^3^ cells/ml), and transferred into a freezing container filled with isopropanol (Thermo Scientific) for a freezing step at − 80 °C until long-term storage in liquid nitrogen. To thaw frozen cells, cryogenic vials were warmed up at room temperature (22–23 °C) until the ice crystals had nearly disappeared. Cell suspension was mixed with triple the volume of culture medium, centrifuged at 130*g* for 5 min, resuspended in cell culture medium, and placed in a T25 flask (TPP) for incubation at 20 °C and 2.5% CO_2_. After the freezing and thawing process, the total number of cells and percentage of viable cells was determined according to the manufacturer’s instructions applying the Countess Automated Cell Counter using trypan blue staining.

Commercially available WF2 cells (IZSLER Brescia) were incubated in 100 mm culture dishes (Sarstedt) with MEM Eagle medium, including Earle’s salts (Sigma) at 20 °C and 3% CO_2_. Medium was complemented with 10% fetal bovine serum (Gibco), 10 mM non-essential amino acids (Merck), 40 mM l-glutamine (Merck), and penicillin/streptomycin (Sigma). Cells were grown until a confluency of ~90% was reached, followed by harvesting for RNA extraction.

### Immunofluorescence

The morphology of SLUlar1 cells at passage six was evaluated by immunofluorescence labeling. 300,000 cells were cultivated in 35-mm μ-dishes (ibidi GmbH) for 24 h, washed three times with PBS, fixed with paraformaldehyde (4%, 10 min, Merck), and permeabilized with Triton X-100 (0.1%, 10 min, Sigma-Aldrich). Actin staining was performed using Bodipy FL Phallacidin (1:40, 30 min, Molecular Probes, Eugene). Focal adhesions were stained using vinculin-Alexa 647 (1:100, 30 min; Abcam, Cambridge, UK), and nuclei were stained by Hoechst 33342 dye (1 μg/ml, Sigma-Aldrich). Finally, cells were analyzed with a confocal laser scanning microscope (LSM) 780 (Carl Zeiss), using a × 63 oil objective via the software ZEN2.3.

### Nucleic acid isolation

For total RNA extraction from different developmental stages of pikeperch, sampled material was homogenized individually in 1 ml TRIzol Reagent (Thermo Fisher Scientific), based on the manufacturer's protocol. SLUlar1 cells (passage six) and WF2 cells (passage 17) were resuspended in 350-μl RLY lysis buffer (ISOLATE II RNA Mini Kit; Bioline) with an additional 3.5 μl of 2-mercaptoethanol (Sigma) following a washing step with phosphate-buffered saline solution (PBS, Biochrom) and centrifugation at 300*g* for 5 min at 15 °C. All samples were subsequently purified with the RNeasy Mini Kit (Qiagen) including DNase treatment. The quality and quantity of isolated RNA were analyzed by agarose gel electrophoresis and spectrophotometry in repeated measurements (ND 1000; NanoDrop Technologies/Thermo Fisher Scientific). RNA was stored at − 80 °C until further use.

### Gene selection and primer design

To establish a screening panel for key steps of the developmental process, we included 21 genes involved in stress (*NR3C1*, endothelial PAS domain protein 1 [*EPAS1*], hypoxia inducible factor 1 subunit alpha [*HIF1A*], heat shock transcription factor 1 [*HSF1*], heat shock transcription factor 2 [*HSF2*], *teleost-specific* osmotic stress transcription factor 1 [*tOSTF1*]; Le Goff et al. 2004; Deane and Woo 2011; Tse 2014; Malandrakis et al. 2016; Pelster and Egg 2018), and immune response (interleukin 1 beta [*IL1B*], lysozyme [*LYZ*]; Saurabh and Sahoo 2008; Zou and Secombes 2016) as well as cell homeostasis (transcription factor EB [*TFEB*]; Settembre and Ballabio 2011; Raben and Puertollano 2016), nutritional status (*APOE*, *PPARA*, *PPARD*; Poupard et al. 2000; Leaver et al. 2005; Napolitano and Ballabio 2016), growth (insulin-like growth factor 2 [*IGF2*], *GHR*; Bergan-Roller and Sheridan 2018; Nipkow et al. 2018), energy metabolism (*CKM*, glycine amidinotransferase [*GATM*]; Borchel et al. 2019), the process of gonadal maturation (*SOX9*; Leet et al. 2011; Bhat et al. 2016), and the organogenesis of the early life stages (*BMP4*, *BMP7*, myosin heavy chain [*MYH6*], *RXRA*; He et al. 2009; Ahi 2016; Bloomquist et al. 2017; Tang et al. 2018) (Table 1). For the stress screening of SLUlar1 and WF2 cells, the additional immune markers interleukin 8 (*CXCL8*) and interleukin 10 (*IL10*) were included. No cDNA sequences were publicly available for the selected candidate genes at the beginning of the study, except for *CXCL8*, and *NR3C1* (Swirplies et al. 2019). Therefore, we identified orthologous gene sequences from the order Perciformes (*Acanthochromis*, *Dicentrarchus*, *Epinephelus*, *Gasterosteus*, *Notothenia*, *Oreochromis*, *Perca*, *Sebastes* spp*.*) from the NCBI (National Centre for Biotechnology Information) GenBank (GB) database. Using BLAST searches against our recently published genome of *S. lucioperca* (RefSeq NCBI: GCA_008315115.1), obtained with Illumina technology and PacBio Sequel System (Nguinkal et al. 2019), we identified the corresponding sequences. To verify the identified sequence fragments, a reciprocal BLAST against the NCBI nucleotide database was performed. Optimal pikeperch-specific oligonucleotide primers (Sigma-Aldrich) were derived using the Pyrosequencing Assay Design software (version 1.0.6; Biotage; Table 1). For primer validation, all PCR products were sequenced on an Applied Biosystems 3500 Genetic Analyzer (Life Technologies).

**Table 1:** Gene-specific primer set used in this study

Gene symbol	Official names	Sense primer (5′–3′)	Antisense primer (3′–5′)	Primer efficiency [%]	Fragment length [bp]
Reference genes					
*EEF1A1*	Elongation factor 1 alpha	ATGGACAGACCCGTGAGCATG	TTCTTGATGTAGGTGCTCACTTC	105	151
*RPL32*	Ribosomal protein L32	GGCGTAAACCCAGAGGTATTGA	ACCTCGAGCTCCTTGACATTGT	105	157
*RPS5*	Ribosomal protein S5	GCAGGATTACATTGCTGTGAAAG	TCATCAGCTTCTTGCCATTGTTG	101	161
Target genes					
Stress response					
*EPAS1*	Endothelial PAS domain protein1	AGTGCAGAGGACGCACAGATG	TCATGTTCACCTGCGTGAGCC	100	139
*HIF1A*	Hypoxia inducible factor 1 subunit alpha	CCAGTCGAATCCCTTGAGAGTT	CTGTGGGGTCCTCTTAGCAAC	97	156
*HSF1*	Heat shock transcription factor 1	TGTGTCTTGTGCAGAGTGGAAC	GCTGGCCATGTTGTTGTGTTTG	111	101
*HSF2*	Heat shock transcription factor 2	AGCCGTCCCGCAGCTCCCT	CGGGACTCAGTTCGCACAGG	91	93
*tOSTF1*	*Teleost-specific* osmotic stress transcription factor 1	CTCCCTTGAATCGGTGGTGAG	GACACTGTGAAAGAAGAGCAGTA	102	109
*NR3C1*	Nuclear receptor subfamily 3 group c member 1	CCAGTCCTGCATGGATTCACTT	AGGTCCATAGTGTTGTCACTGAA	100	180
Immune response					
*CXCL8*†	Interleukin 8	AACAGGGATGAGTCTGAGAAGC	GCTTGGAAATGAAGTCTTACATGA	98	158
*IL1B*	Interleukin 1 beta	TCGACCTACTTGCACCCTACA	TCTGCCTCCACAACCTGAA	101	137
*IL10*†	Interleukin 10	TTTGCCTGCCACGCCATGAAC	AGGCTTTAAGTCATTGGTCTCCT	95	102
*LYZ*	Lysozyme	TTTGGCCAACGCCAGGGTCTA	TCCGTCTGTGTTGTGGTTGATG	98	160
Cell homeostasis					
*TFEB*	Transcription factor EB	AGTGATGTGCGCTGGAACAAAG	CCTGTTACCTGGATGCGTAGC	95	158
Nutritional status					
*APOE*	Apolipoprotein E	GCTAGAGCACTCTGATCTCTGA	TTGGCATCCAGCATGTCCTTCT	99	160
*PPARA*	Peroxisome proliferator-activated receptor alpha	ATCTGAATGATCAGGTGACTCTC	TTGGGCTCCATCATGTCGCTAA	96	172
*PPARD*	Peroxisome proliferator-activated receptor delta	CTTTGTGACCAGGGAGTTCCTT	AGGACGATCTGGACAGAGAATAA	99	157
Growth					
*GHR*	Growth hormone receptor	ACCACAAACTGGGAAGCATTGGA	CCTTTGCTGGGAATCTCAGTCA	96	173
*IGF2*	Insulin-like growth factor 2	GAGGCTTCTATTTCAGGTAGGC	ACGGGTATGACCTGCAGAGAG	108	179
Energy metabolism					
*CKM*	Ceratine kinase, M	AGTACTACCCCCTGAAGTCCAT	TCTTGCTGTCGTTGTGCCAGAT	98	156
*GATM*	Glycine amidinotransferase	ATCCTTCTGGTTGTCGGGAATG	GGATGGGGTAGTCCTGAACATA	92	178
Gonadal maturation					
*SOX9*	SRY-box transcription factor 9 c	CGCGTTAACGGCTCAAGTAAAAA	TTCGTTGAGCAATCTCCAAAGTTT	94	165
Organogenesis					
*BMP4*	Bone morphogenetic protein 4	CCGTAAACGCAACCGCAACTG	TGAGTTCAGATGATCCGCCAGA	94	151
*BMP7*	Bone morphogenetic protein 7	TGTTTCTGCTGGACTCTCGGG	TTGATGCTCTCTCCGTTTGTGC	98	151
*MYH6*	Myosin heavy chain	GGGAAGACTGTGAACACCAAGA	TCCCGAAGCGAGACGAGTTGT	98	175
*RXRA*	Retinoid x receptor alpha	CATGAAGAGAGAAGCCGTTCAG	GTATGTCTCGGTTTTGGGTTCC	98	151

^†^Genes applied exclusively for in vitro analysis

### Real-time quantitative PCR

For gene expression profiling in vivo and in vitro, similar as in our previous study (Swirplies et al. 2019), real-time quantitative PCR (RT-qPCR) was performed with a LightCycler96 system (Roche Diagnostics) and the SensiFAST™ One-Step qPCR kit (Bioline), in line with the manufacturer’s instructions. Therefore, cDNA synthesis from 1.5 μg (in vivo samples) or 0.03–0.1 μg (in vitro samples) of total RNA was performed using the SuperScript II Reverse Transcriptase Kit (Thermo Fisher Scientific) based on the manufacturer's instructions. The resulting cDNA was stored at − 20 °C until further use. An initial denaturation step (95 °C, 5 min) was followed by 40 cycles of denaturation (95 °C, 5 min), 15 s of annealing (60 °C), 15 s of elongation (72 °C), and a fluorescence measurement step for 10 s (72 °C). Standard curves were established for all genes to calculate the copy numbers using linear regression analysis (*R*_2_ > 0.998). These were based on the *C*_q_ values of tenfold dilutions of the generated fragments (1 × 10^3^ –1 × 10^8^ copies). *C*_q_ values < 35 were considered detectable. For data normalization, three reference genes (*EEF1A1, RPL32, RPS5*) which were already established for data normalization in pikeperch (Swirplies et al. 2019) were included and evaluated for each sample (Table 1). For quality control, PCR products were verified via gel electrophoresis and melting curve analysis.

### Statistics

RT-qPCR data were analyzed with the LightCycler 96 analysis software v.1.1, and the suitability of reference genes was assessed using the qBase+ software (Biogazelle, with CV ≤ 0.3). The statistical significances of different ontogenesis stages of pikeperch were calculated using the one-way analysis of variance (ANOVA) followed by parametric Tukey’s multiple comparison test using the GraphPad Prism 8 software, version 8.3.0.538. To analyze cell number and viability, mean and S.E.M. were calculated for all cell passages of SLUlar1.

## Results

Ontogenetic stage-specific expression profiles of unchallenged pikeperch

**Fig. 1:**
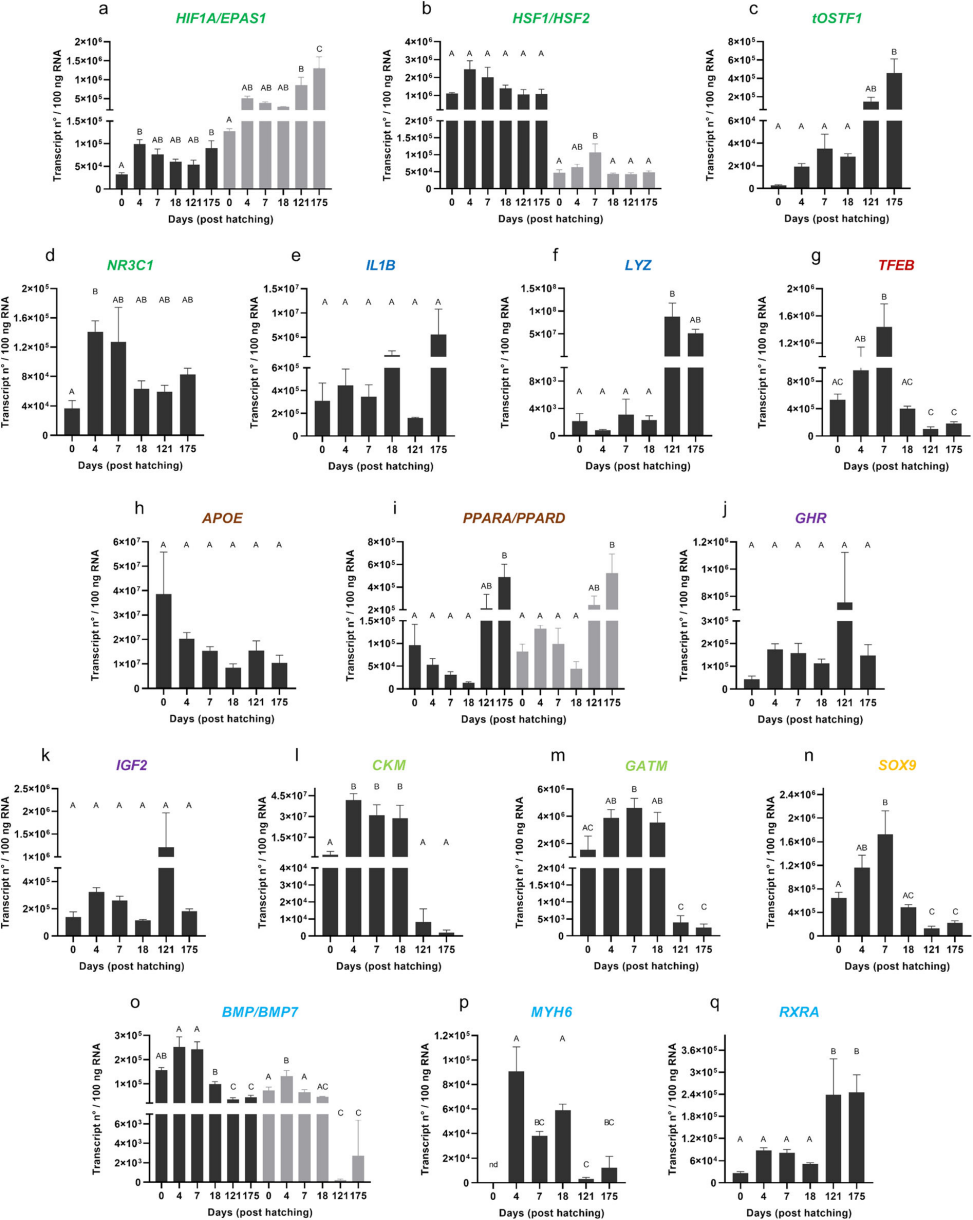
Transcription patterns of candidate genes in developing pikeperch. Genes categorized in stress response (dark green), immune response (dark blue), cell homeostasis (red), nutritional status (brown), growth (purple), energy metabolism (light green), maturation (yellow), and organogenesis (light blue). Columns represent normalized mean (+SEM), calculated per 100 ng of total RNA of each three pools of eyed eggs (0 days post hatch (dph); *n* = 20/pool), yolk sac larvae (4 dph; n = 20/pool), larvae fed with *Artemia* spp*.* (7 dph; *n* = 30/pool), larvae fed with dry feed (18 dph; n = 30/pool), and three individual samples of liver tissue from fingerlings (121 and 175 dph). Different letters (A–D) indicate significant changes in transcript numbers (*p* < 0.05); in the case of two represented genes: first depicted in black and second depicted in grey. nd: no data detectable

In the current study, we defined the basal mRNA abundance of the 21 genes listed in Table 1 at 0 dph, 4 dph, 7 dph, 18 dph, 121 dph, and 175 dph of farmed pikeperch through RT-qPCR analysis (Fig. 1). The average transcript numbers of the analyzed genes ranged from around 1 × 10^1^ (*BMP7* at 121 dph) to 1 × 10^8^ (*LYZ* at 121 dph) copies per 100 ng RNA. We detected ontogenesis-specific transcription patterns for all genes analyzed with the exception of *HSF1*, which was constitutively expressed. For *MYH6*, we did not obtain valid data at 0 dph.

For 10 genes, we observed the highest copy numbers in early developmental stages (0–18 dph). *APOE* reached the highest transcript levels at 0 dph. The transcript levels of *BMP4*, *BMP7*, *CKM*, *MYH6*, and *NR3C1* were highest at 4 dph. While the expression of *BMP4* significantly decreased from early (4 and 7 dph) to late larval (18 dph) and fingerling stages (121 and 175 dph), that of *BMP7*, *CKM*, and *NR3C1* significantly increased from 0 dph to either 4 dph (*BMP7* and *NR3C1*) or all larval stages (4–18 dph; *CKM*). The four genes *GATM*, *HSF2*, *SOX9*, and *TFEB* were most strongly expressed at 7 dph, whereby the transcript levels of *HSF2*, *SOX9*, and *TFEB* significantly increased between 0 and 7 dph*. GATM* significantly decreased between the early (0–18 dph) and late developmental stages (121 and 175 dph).

The genes *GHR*, *IGF2*, and *LYZ* were most strongly expressed at 121 dph, whereby the expression of *LYZ* was highly significantly (*p* = 0.003) increased from early (0–18 dph) to late developmental stages (121 dph). *EPAS1*, *IL1B*, *tOSTF1*, *RXRA*, *PPARA*, and *PPARD* revealed the highest transcript levels at the fingerling stage of 175 dph. Thereby, *EPAS1* and *RXRA* were significantly increased between the early (0–18 dph) and late developmental stages (121 and 175 dph). The expression of *tOSTF1*, *PPARA*, and *PPARD* was enhanced compared with the early developmental stages but only significant at 121 dph.

*HIF1A* significantly increased in transcript number from 0 to 4 dph as well as the late fingerling stage (175 dph), with similarly high copy numbers for each.

### Characterization of the basal stress level of a long-lasting primary cell culture

**Fig. 2:**
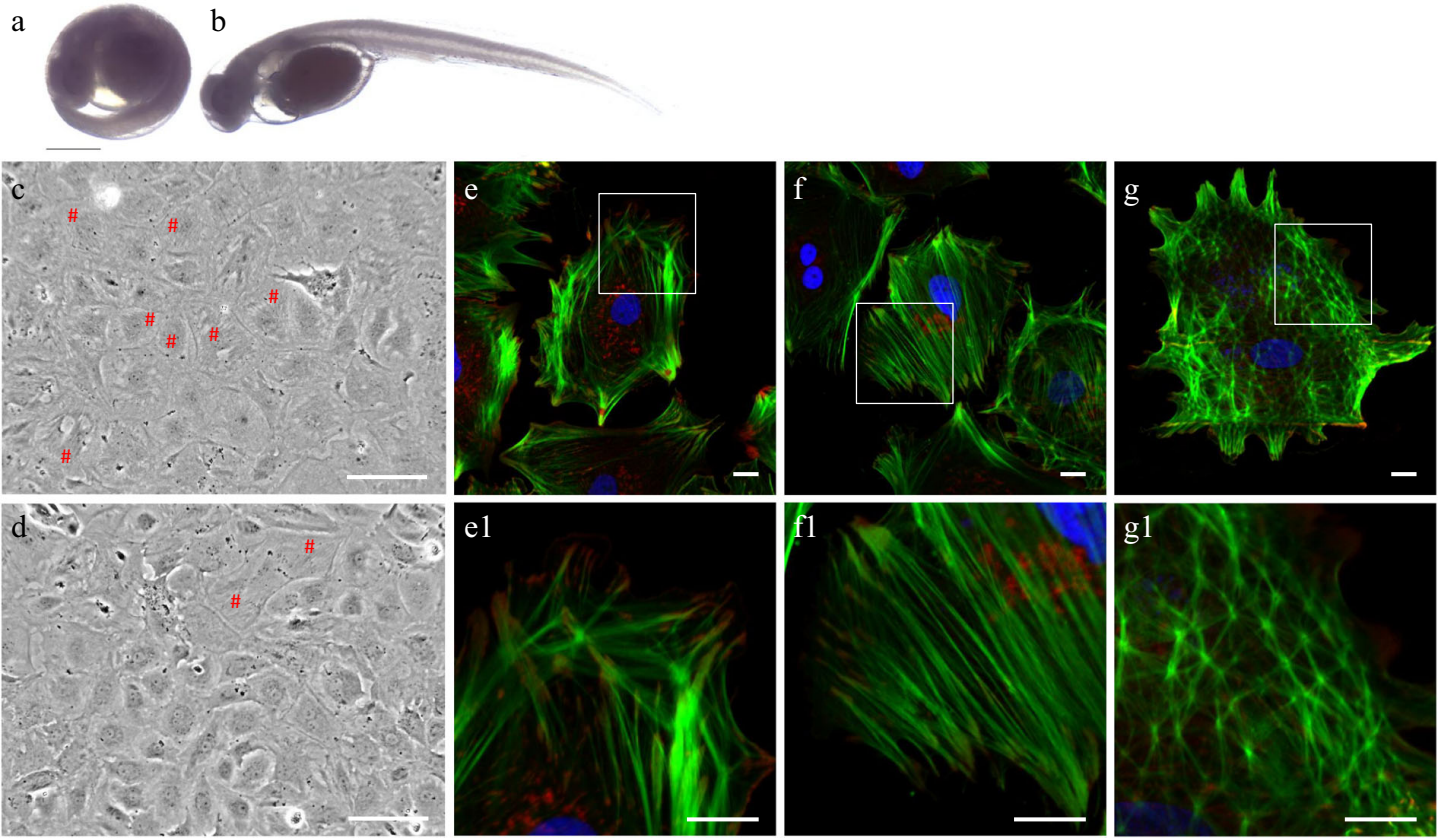
Source and morphology of SLUlar1 cells from *Sander lucioperca*. Larvae at an age of 125 degree days (DD; nine days after fertilization) in (a) the egg and (b) isolated from the eggshell, phase contrast microscopy of isolated SLUlar1 cells from (c) passage five and (d) passage six. High concentrations of actin filaments are marked with #. Immunofluorescence: Cells with cortical actin rings (e) clearly differ from cells with stress fibers throughout the cell body (f) and cells with cross-linked actin networks (g). ß-actin: green; vinculin: red; nuclei: Hoechst 33342 dye. Scale bars: (a–d) 100 μm, (e–g) 10 μm

As the first approach towards a cell model from pikeperch, the long-lasting primary cell culture SLUlar1 out of isolated embryos was established (Fig. 2a, b). Following cell isolation, single cells and tissue fragments attached to the culture dish within 48 h. With increasing numbers of passages, the cells grew in monolayer, and from the third passage onwards, tissue fragments were no longer present. Cell size increased from 10 to 13 μm in passage two to 15 to 17 μm in passage six. Cells exhibited a density of 7.5 × 10^5^ ± 0.4 × 10^3^ cells/ml with a vitality of 91 ± 2% after trypsinization. After thawing, cell vitality was around 89 ± 0.7%. However, even with a vitality of 90%, only 60 to 70% of the cells attached to the bottom after 2 days. Therefore, cells needed up to 4 weeks for recovery and to reach a confluency of 80 to 90%. Although we tested various cultivation conditions, including different temperatures (16 °C, 20 °C, and 25 °C), different cell culture media (DMEM and Leibowitz-15), as well as gas mixtures (with or without CO_2_), cells stopped proliferating at passage eight and remained in the stagnation phase but without signs of cell death.

The morphology of SLUlar1 cells at passage six was evaluated by phase-contrast microscopy (Fig. 2c, d) and immunofluorescence labeling of β-actin and vinculin (Fig. 2e–g). A high proportion of stress fiber formations and cross-linked actin networks (CLANs) was observable. Regarding the location of β-actin, three different population types were determined: (i) cells with well-established cortical actin rings in the periphery and rather thin actin fibers spanning the cell body (Fig. 2e, e1), (ii) cells with strong stress fiber formation throughout the cell body (Fig. 2f, f1), and, frequently observed, (iii) cells with actin arranged in a net-like shape as it is described for CLANs (Fig. 2 g, g1). The focal adhesions (represented by vinculin staining) of cells with cortical actin were located in the cell margins and larger than cells with stress fibers. In contrast, cells with CLANs exhibited high numbers of rather small focal adhesion spots throughout the entire cell.

**Fig. 3:**
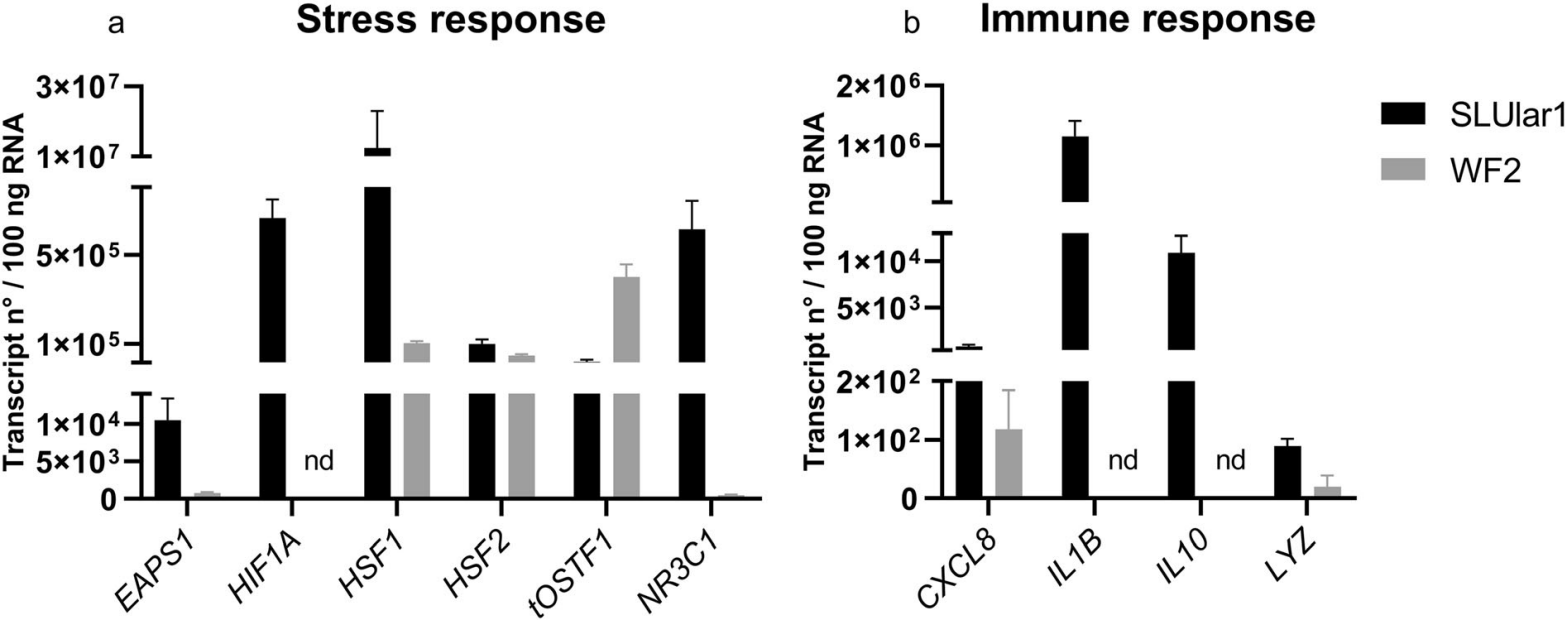
Basal transcript levels of stress and immune marker in unchallenged cell models. Transcription patterns of genes important for stress (a) and immune response (b) in SLUlar1 (black columns) and WF2 cells (grey columns). Columns represent normalized means of three individual samples (+ SEM), calculated per 100 ng of total RNA. nd: no data detectable

Since this altered cell morphology indicates stress, we further investigated the stress level of the cells and compared it to that of the established cell line WF2 by determining the transcript level of 10 marker genes for stress (*HIF1A*, *EPAS1*, *HSF1*, *HSF2*, *tOSTF1*, and *NR3C1*) and immunity (*LYZ*, *IL1B*, *CXCL8*, and *IL10*) at cell passage six (Fig. 3). In SLUlar1 cells, we detected high transcript levels of *HSF1* (1 × 10^7^) and *IL1B* (1 × 10^6^), moderate mRNA levels of *HIF1A* (7 × 10^5^), *NR3C1* (6 × 10^5^), *HSF2* (1 × 10^5^), and *tOSTF1* (2 × 10^4^), as well as *EPAS1* (1 × 10^4^), and low transcript numbers for *CXCL8* (8 × 10^2^) and *LYZ* (9 × 10^1^). In WF2 cells, the highest copy numbers were detected for *tOSTF1* (4 × 10^5^), *HSF1* (1 × 10^5^), and *HSF2* (5 × 10^4^), while *EPAS1* (8 × 10^2^), *NR3C1* (5 × 10^2^), *CXCL8* (1 × 10^2^), and *LYZ* (6 × 10^1^) were only marginally expressed. Transcript levels of *HIF1A*, *IL1B*, and *IL10* were not detectable.

### Expression profiles of unchallenged WF2 cell line

Due to the above described difficulties in establishing a specific cell line from *S. lucioperca*, we investigated the suitability of the existing cell line WF2 from *S. vitreus* to examine the expression of the 21 ontogenetic target genes from the in vivo study (compare Table 1). For 16 of these genes, we detected relevant transcript numbers in WF2 cells. However, we did not for *CKM*, *IGF2*, *HIF1A*, *IL1B*, and *GATM*. We recorded average transcript numbers from 6 × 10^1^ (*LYZ*) to 2×10^6^ (*BMP4*) copies per 100 ng RNA (Table 2). Gene expression in the WF2 cell line was similar compared with that in pikeperch at 0 dph in the case of *GHR*, at 4 dph in the case of *PPARD*, and fingerlings in the case of *MYH6* (175 dph), *PPARA* (175 dph), *RXRA* (121 and 175 dph), and *tOSTF1* (121 and 175 dph). Moreover, transcript levels of *HSF2* in the cells were similar to several developmental stages of whole pikeperch (0, 18, 121, 175 dph). In contrast, *APOE*, *TFEB*, *GATM*, *EPAS1*, *HSF1*, *NR3C1*, *LYZ*, and *SOX9* were expressed slightly less, while *BMP4* and *BMP7* were more strongly expressed in WF2 cells than in vivo.

**Table 2:** Expression profiles of candidate genes in the cell line WF2

	*EPAS1*	*HSF1*	*HSF2*	*tOSTF1*	*NR3C1*	*LYZ*	*TFEB*	*APOE*	*PPARA*	*PPARD*	*GHR*	*SOX9*	*BMP4*	*BMP7*	*MYH6*	*RXRA*
n°	7.76E+02	1.03E+05	4.77E+04	4.00E+05	4.89E+02	5.92E+01	4.05E+04	1.80E+04	5.29E+05	1.57E+05	6.67E+04	2.00E+04	1.54E+06	7.26E+05	1.60E+04	2.32E+05
SEM	1.02E+02	7.40E+03	4.86E+03	4.54E+04	7.78E+01	1.61E+01	5.92E+03	1.24E+03	1.09E+04	8.96E+03	3.11E+03	1.36E+03	1.56E+05	5.34E+04	6.02E+03	1.71E+04

*n°*, transcript number per 100 ng of total RNA; *SEM*, standard error of the mean; not detectable: *CKM*, *GATM*, *HIF1A*, *IGF2*, *IL1B*

## Discussion

The farming of pikeperch in intensive aquaculture systems such as RAS is continuously growing in importance for European aquaculture. The strategies for intensive pikeperch rearing generally focus more on economic considerations than on welfare concerns. However, optimizations of intensive aquaculture techniques are necessary to improve animal welfare as well as to increase the production of fingerlings for stocking considering the determination of optimal breeding parameters as well as the definition of limit values. The current study evaluated key genes involved in the early steps of ontogenesis to improve basic knowledge of the physiological processes during pikeperch rearing and the associated challenges.

### Transcription factors *HSF1* and *HIF1A* with uniform or biphasic expression patterns

While the majority of the examined genes displayed an ontogenesis-specific transcription pattern with a peak expression at a specific developmental stage, *HSF1* was constitutively expressed. This transcription factor is the major regulating factor involved in the response to environmental stressors in vertebrates (Morimoto 1998; Buckley and Hofmann 2002; Padmini and Usha Rani 2009). It coordinates the transcriptional activation of heat shock proteins (HSPs), protects protein and lipid metabolism during stress conditions, and contributes to distinct immune processes (Deane and Woo 2004; Roberts et al. 2010). The constant expression of *HSF1* in the present study indicates that no specific stress response was induced by *HSF1* at any time point.

For *HIF1A*, we noticed a biphasic expression pattern with a significant increase of copy numbers at 4 and 175 dph compared with the transcript level at 0 dph. During organogenesis as well as during the period of growth in general, the organs require higher levels of oxygen (Anderson and Podrabsky 2014). *HIF1A* is the major regulator of the response to hypoxia (Rytkönen et al. 2007; Geng et al. 2014). Additionally, *HIF1A* is involved in several physiological processes during vertebrate development, such as angiogenesis, glucose uptake and metabolism, and cellular proliferation, as well as apoptosis (Gracey et al. 2001; Vuori et al. 2004; Rojas et al. 2007; Liu et al. 2017; Tan et al. 2017). The observed constitutive *HIF1A* expression pattern in pikeperch resembles observations during the early ontogenesis of zebrafish (*Danio rerio*), Wuchang bream (*Megalobrama amblycephala*), and lake whitefish (*Coregonus clupeaformis*) (Rojas et al. 2007; Shen et al. 2010; Whitehouse and Manzon 2019). Nevertheless, *HIF1A* appears to underly species-specific modulations during the different developmental stages, as exhibited in zebrafish and lake whitefish (Rojas et al. 2007; Whitehouse and Manzon 2019).

### Gene expression of *APOE*, *BMP4*, *BMP7*, *CKM*, *GATM*, and *TFEB* reflect challenges in feed conversion

At 0 dph, we uncovered high *APOE* transcript levels. The encoded apolipoprotein E is involved in the vertebrate lipid metabolism, where it is crucial for the internalization of plasma lipoproteins into the cell (Mahley 1988; Babin et al. 1997). Fish egg yolk contains high amounts of lipoproteins (Wiegand 1996). Poupard et al. (2000) determined high levels for *APOE* transcripts in the yolk sac of embryonic and larval turbot (*Scophthalmus maximus*). *APOE* is highly expressed in the yolk syncytial layer and appears to control its utilization during the early steps of ontogenesis (Otis et al. 2015). Thus, our findings in pikeperch embryos are in line with this study. Furthermore, our data indicate a general decline in the expression levels from 0 dph to the later stages, suggesting the necessity of *APOE* for the continuous consumption of the yolk sac reserves from egg to larvae as well as the final basal expression level for lipid metabolism after entering the exotrophic feeding phase.

The examined yolk sac larvae at 4 dph demonstrated the highest expression levels for genes involved in organogenesis (*BMP4*, *BMP7*, and *MYH6*), general stress response (*NR3C1*), and energy metabolism (*CKM*). *BMP4* and *BMP7* encode for bone morphogenetic proteins 4 and 7. These proteins are involved in the process of chondro- and skeletogenesis as well as in the morphogenesis of several vertebrate organs (Streelman et al. 2003; Hoffman et al. 2006; Adams et al. 2007; Bonilla-Claudio et al. 2012). *MYH6* is expressed in the heart muscles of vertebrates (Dhillon et al. 2009; López-Unzu et al. 2019). The myosin heavy chain is the major component of the motor protein myosin in eukaryotic cells (Vikstrom et al. 1997).

*Perciformes* such as pikeperch and Eurasian perch undergo several morphological changes, including skull and jaw adaptation as well as fin and teeth development around hatching time and until the start of the first uptake of exogenous feed (Löffler et al. 2008; Ott et al. 2012b; Alix et al. 2015). Güralp et al. (2017) determined the beginning of pectoral fin formation at 1 dph and mouth opening at 5 dph in pikeperch reared at 15 °C (Güralp et al. 2017). Before mouth opening, Ostaszewska et al. (2005) observed changes within the larval intestines of pikeperch, including the length, the lumen, and the appearance of the mucosal lining. Another aspect of organogenesis is the growth of the developing organs (Ostaszewska 2005). The first heart beating in pikeperch was observed at the 34-somite stage (around 1.5 h post fertilization [hpf]) and the beginning of blood circulation at the 50-somite stage, right before the start of hatching (equal to around 2 hpf) (Güralp et al. 2017). In our study, the highest expression of the *BMP4* and *MYH6* at 4 dph might reflect these processes. In line with this, the subsequent stages of fed larvae and fingerlings were characterized by a significant decrease in copy numbers (except for *BMP4* at 7 dph).

The process of hatching and adapting to new environmental conditions is stressful and therefore energy-consuming. The glucocorticoid receptor, encoded by *NR3C1*, is the main regulator of the general stress response in vertebrates, including developing fish (Pavlidis et al. 2011; Tsalafouta et al. 2018). We recorded a significant *NR3C1* increase from 0 to 4 dph, with a stable expression at later stages of ontogenesis. CKM serves as an energy buffer in mammals and fish (Wyss and Kaddurah-Daouk 2000; Borchel et al. 2014; Borchel et al. 2019). It is responsible for the dephosphorylation of creatine in muscle cells, which is further used for the regeneration of ADP to ATP within the target tissue (Wyss and Kaddurah-Daouk 2000). Our results for *NR3C1* and *CKM* seem to reflect the restructuring programs of the developing body of freshly hatched larvae and the challenges of the new environment such as oxygen and carbon dioxide gas exchange or the acclimatization to new energy resources due to exogenous feed intake (*Artemia* spp. followed by dry feed).

At day 5 post hatching, the mouth opens and the mixotrophic feeding phase begins, until complete yolk sac resolution (up to 14 dph) (Güralp et al. 2017). Due to the small size of freshly hatched pikeperch (4–5-mm total length) (Schlumberger and Proteau 1996) and the incompletely developed intestine, the initial feeding requires live prey (Hamza et al. 2007). The phase of conversion from endo- to exotrophic feeding is highly critical, since this is when fish react strongly to chemical or physical stimuli (Woltering 1984). During this important phase of development at 7 dph, genes involved in energy metabolism (*GATM*), nutrition (*TFEB*), gonadal maturation (*SOX9*), and stress response (*HSF2*) were most strongly expressed.

GATM contributes to the creatine energy system in mammals and fish (Borchel et al. 2014; Borchel et al. 2019). For pikeperch, we detected similar expression levels in all larval stages at 4–18 dph, with the highest copy numbers at 7 dph. At that stage, larvae experience exogenous feed intake including digestion for the first time. Moreover, the complete yolk sac resorption must be compensated to reach homeostasis of the energy metabolism. Thus, high expression of *GATM* is concordant with the required new energy levels. In line with this, we found that the expression of the gene encoding transcription factor EB (*TFEB*) gradually increased from 0 dph to larvae fed with *Artemia* spp at 7 dph. and then decreased in later developmental stages. In vertebrates, TFEB is important for cell homeostasis and is involved in several cellular processes, such as lipid metabolism, bone resorption, and immune response (Ferron et al. 2013; Settembre et al. 2013; Tiller and Garsin 2014). According to Settembre et al. (2013), TFEB is the main factor coordinating the metabolic response to the process of starvation in the nematode *Caenorhabditis elegans*. The first oral feeding most likely provoked an immune response due to the first contact with non-self molecules. Furthermore, the final resorption of the endogenous yolk sac energy resources might lead to a state similar to starvation (until first feeding), while the exogenous feed intake delivers energy. All three conditions might modulate the observed transcription of *TFEB*.

SOX9 is critical for the sex determination, gonad formation, and development of vertebrates (Yokoi et al. 2002; Chaboissier et al. 2004). In Nile tilapia (*Oreochromis niloticus*), *SOX9* is highly expressed during the early ontogenesis of both sexes, but the concentration decreases in later stages (Ijiri et al. 2008). In pikeperch, we found a similar transcription pattern with the highest expression of *SOX9* at 7 dph, followed by a decline in later developmental stages. This pattern was also observed for *HSF2*, which is involved in the development of embryos in vertebrates (Eriksson et al. 2000). This pattern might reflect the contribution of both factors to early developmental processes.

Subsequent to the transition from endo- to exotrophic feeding, the conversion to artificial feed is another challenging phase that influences growth and mortality (Kestemont et al. 2007; Hubenova et al. 2015). Several studies have demonstrated that the transversion from endo- to exogenous feed is a critical step in the farming of fish larvae (Hamza et al. 2007; Kestemont et al. 2007). Unexpectedly, none of the evaluated genes displayed a peak expression in the stage of larvae fed with dry feed (18 dph).

### The genes *GHR*, *IGF2*, and *LYZ* were highly expressed during the growth phase of juvenile stages

Within fingerlings at 121 and 175 dph, genes of stress (*tOSTF1*, *EPAS1*) and immune response (*LYZ, IL1B*), growth (*GHR*, *IGF2*), organogenesis (*RXRA*), and nutritional status (*PPARA*, *PPARD*) were highly expressed. The innate immune genes *LYZ* and *IL1B* are part of the first line of defense. The transcript levels of c- and g-type lysozyme were found to be low in olive flounder (*Paralichthys olivaceus*) from hatching until 20 dph, followed by a significant increase to 50 dph (Lee et al. 2014). Our results indicate a similar significant increase in the mRNA levels of *LYZ* from early stages at 0–18 dph to the stage of juveniles at 121 dph.

The two genes, *GHR* and *IGF2*, are involved in the growth of fish (Schlueter et al. 2007; Besseau et al. 2013; Claudino da Silva et al. 2019). Investigations of gilthead sea bream (*Sparus aurata*) demonstrated a positive correlation between the expression of *GHR* and a growth spurt during summer months (Calduch-Giner et al. 2003). Nipkow et al. (2018) detected increased *IGF2* transcript levels in maraena whitefish (*Coregonus maraena*) at the onset of oral feeding and during development into fingerlings. We found similar patterns in pikeperch with an increase in *IGF2* and *GHR* transcript levels from 0 dph to 4 and 7 dph and the highest levels at 121 dph, reflecting the strong phase of growth of fingerlings.

### The long-lasting primary cell culture SLUlar1 shows prominent sensitivity including stress fiber formation and the high expression of stress-related genes

To establish optimal pikeperch farming conditions, detailed research regarding its physiological needs is indispensable. Especially in basic research, in vitro analyses can be a suitable replacement for animal experiments. Currently, no specific cell model of pikeperch is available.

Here, we present an approach to derive a cell model from pikeperch. However, embryonal *Sander* cells are apparently more sensitive to the standard handling process compared with other primary fish cells from, for example, Atlantic sturgeon, Atlantic salmon (*Salmo salar*), Siberian sturgeon (*Acipenser baerii*), maraena whitefish, rainbow trout (*Oncorhynchus mykiss*), and zebrafish (Ciba et al. 2008; Grunow et al. 2011b; Grunow et al. 2011a; Grunow et al. 2015). The cells stopped to proliferate and remained in the stagnation phase from passage eight onward. Furthermore, they accumulated actin filaments in the cytoskeleton, which are well-known indicators of stress. We identified three different actin populations at cell passage six; one had cortical actin rings and low stress fiber formation, which might be derived from cells of the epithelial or endothelial lineage. These cells grew firmly together and formed a stable cell architecture by using their neighboring cells as mechanical support. In another population we observed significant stress fibers, which characterizes mechanically stretched cells like muscle cells or bone cells. These cells transmit forces through their cell body and therefore have a strong actin network. Moreover, we observed a star-shaped formation of actin, which has not been described in unstimulated cells thus far. However, CLANs (cross-linked actin networks) can occur under stress or by stimulating different integrin signaling pathways (i.e., via different extracellular matrix proteins) (Filla et al. 2009; Job et al. 2010).

On the transcriptional level, high transcript numbers were detected for immune and stress marker genes *IL1B*, *NR3C1, HIF1A*, *HSF1*, and *HSF2* at the sixth cell passage. *IL1B* is a well-established marker for in vitro stimulation with pathogen-associated molecular patterns (PAMPs) in the primary cells of different fish species (Chaves-Pozo et al. 2004; Martorell Ribera et al. 2020). Compared with the cell line WF2, SLUlar1 cells showed prominent higher transcript levels for the examined genes, except for *tOSTF1*. Along with high expression levels of either general cellular and environmental (*NR3C1*) or specific environmental stress response genes (*HIF1A*, *HSF1*, *HSF2*), we conclude that the current culturing conditions lead to induced stress within the SLUlar1 cells.

### The WF2 cell line is currently the most suitable cell model, but does not correspond to a specific ontogenetic stage of pikeperch

As a substitution for a cell model from *S. lucioperca*, we initially tested the applicability of the in vitro system WF2 (*S. vitreus*) for investigating developmental processes of pikeperch. The expression for most of the genes could be verified, although a clear assignment to a certain developmental stage of the investigated pikeperch samples could not be detected. However, we must be aware of the dissimilarity between an in vitro model and the complexity of a whole organism. Moreover, no further detailed information about the exact ontogenetic stage of the source material is available.

## Conclusion

The process of ontogenesis is accompanied by the continuous adaptation to changing physiological and environmental conditions. In the present study, we determined basal expression patterns of promising molecular markers for monitoring the developmental process of early ontogenesis in pikeperch under current farming conditions. We identified promising candidates representing the challenging steps of feed conversion (*APOE*, *BMP4*, *BMP7*, *CKM*, *GATM*, and *TFEB*) and the growth phase of juvenile pikeperch (*GHR*, *IGF2*, *RXRA*, *PPARA*, and *PPARD*), which can be used to accompany the development process of pikeperch farming in future studies. A first approach to establishing a long-lasting primary cell culture from whole pikeperch embryos was achieved. However, the importance of establishing a suitable cell line has been demonstrated, since it remains a major challenge to yield reproducible results.

## Data Availability

Not applicable.

## References

[CR1] Adams D, Karolak M, Robertson E, Oxburgh L (2007). Control of kidney, eye and limb expression of Bmp7 by an enhancer element highly conserved between species. Dev Biol.

[CR2] Ahi EP (2016). Signalling pathways in trophic skeletal development and morphogenesis: Insights from studies on teleost fish. Dev Biol.

[CR3] Alix M, Chardard D, Ledoré Y, Fontaine P, Schaerlinger B (2015). An alternative developmental table to describe non-model fish species embryogenesis: Application to the description of the Eurasian perch (*Perca fluviatilis* L. 1758) development. Evodevo.

[CR4] Anderson SN, Podrabsky JE (2014). The effects of hypoxia and temperature on metabolic aspects of embryonic development in the annual killifish *Austrofundulus limnaeus*. J Comp Physiol B.

[CR5] Babin PJ, Thisse C, Durliat M, Andre M, Akimenko MA, Thisse B (1997). Both apolipoprotein E and A-I genes are present in a nonmammalian vertebrate and are highly expressed during embryonic development. Proc Natl Acad Sci U S A.

[CR6] Baekelandt S, Redivo B, Mandiki SNM, Bournonville T, Houndji A, Bernard B, El Kertaoui N, Schmitz M, Fontaine P, Gardeur JN, Ledoré Y, Kestemont P (2018). Multifactorial analyses revealed optimal aquaculture modalities improving husbandry fitness without clear effect on stress and immune status of pikeperch *Sander lucioperca*. Gen Comp Endocrinol.

[CR7] Baekelandt S, Mandiki SNM, Schmitz M, Kestemont P (2019). Influence of the light spectrum on the daily rhythms of stress and humoral innate immune markers in pikeperch *Sander lucioperca*. Aquaculture.

[CR8] Baekelandt S, Milla S, Cornet V, Flamion E, Ledoré Y, Redivo B, Antipine S, Mandiki SNM, Houndji A, El Kertaoui N, Kestemont P (2020). Seasonal simulated photoperiods influence melatonin release and immune markers of pike perch *Sander lucioperca*. Sci Rep.

[CR9] Bergan-Roller HE, Sheridan MA (2018). The growth hormone signaling system: Insights into coordinating the anabolic and catabolic actions of growth hormone. Gen Comp Endocrinol.

[CR10] Besseau L, Fuentès M, Sauzet S, Beauchaud M, Chatain B, Covès D, Boeuf G, Falcón J (2013). Somatotropic axis genes are expressed before pituitary onset during zebrafish and sea bass development. Gen Comp Endocrinol.

[CR11] Bhat IA, Rather MA, Saha R, Pathakota GB, Pavan-Kumar A, Sharma R (2016). Expression analysis of Sox9 genes during annual reproductive cycles in gonads and after nanodelivery of LHRH in *Clarias batrachus*. Res Vet Sci.

[CR12] Bloomquist RF, Fowler TE, Sylvester JB, Miro RJ, Streelman JT (2017). A compendium of developmental gene expression in Lake Malawi cichlid fishes. BMC Dev Biol.

[CR13] Bonilla-Claudio M, Wang J, Bai Y, Klysik E, Selever J, Martin JF (2012). Bmp signaling regulates a dose-dependent transcriptional program to control facial skeletal development. Development.

[CR14] Borchel A, Verleih M, Rebl A, Kühn C, Goldammer T (2014). Creatine metabolism differs between mammals and rainbow trout (*Oncorhynchus mykiss*). Springerplus.

[CR15] Borchel A, Verleih M, Kühn C, Rebl A, Goldammer T (2019). Evolutionary expression differences of creatine synthesis-related genes: Implications for skeletal muscle metabolism in fish. Sci Rep.

[CR16] Buckley BA, Hofmann GE (2002). Thermal acclimation changes DNA-binding activity of heat shock factor 1 (HSF1) in the goby *Gillichthys mirabilis*: Implications for plasticity in the heat-shock response in natural populations. J Exp Biol.

[CR17] Calduch-Giner JA, Mingarro M, Vega-Rubín De Celis S, Boujard D, Pérez-Sánchez J (2003). Molecular cloning and characterization of gilthead sea bream (*Sparus aurata*) growth hormone receptor (GHR). Assessment of alternative splicing. Comp Biochem Physiol B Biochem Mol Biol.

[CR18] Chaboissier MC, Kobayashi A, Vidal VIP, Lützkendorf S, van de Kant HJG, Wegner M, de Rooij DG, Behringer RR, Schedl A (2004). Functional analysis os Sox8 and Sox9 during sex determination in the mouse. Development.

[CR19] Chaves-Pozo E, Pelegrín P, García-Castillo J, García-Ayala A, Mulero V, Meseguer J (2004). Acidophilic granulocytes of the marine fish gilthead seabream (*Sparus aurata* L.) produce interleukin-1β following infection with Vibrio anguillarum. Cell Tissue Res.

[CR20] Ciba P, Schicktanz S, Anders E, Siegl E, Stielow A, Klink E, Kruse C (2008). Long-term culture of a cell population from Siberian sturgeon (*Acipenser baerii*) head kidney. Fish Physiol Biochem.

[CR21] Claudino da Silva SC, Lala B, de Carniatto CHO, Schamber CR, Nascimento CS, Braccini GL, Porto C, Roldi G, Tanamati F, Gasparino E (2019). Fumonisin affects performance and modulates the gene expression of IGF-1 and GHR in Nile tilapia fingerlings and juveniles. Aquaculture.

[CR22] Deane EE, Woo NYS (2004). Differential gene expression associated with euryhalinity in sea bream (*Sparus sarba*). Am J Phys Regul Integr Comp Phys.

[CR23] Deane EE, Woo NYS (2011). Advances and perspectives on the regulation and expression of piscine heat shock proteins. Rev Fish Biol Fish.

[CR24] Dhillon RS, Esbaugh AJ, Wang YS, Tufts BL (2009). Characterization and expression of a myosin heavy-chain isoform in juvenile walleye *Sander vitreus*. J Fish Biol.

[CR25] Eriksson M, Jokinen E, Sistonen L, Leppa S (2000). Heat shock factor 2 is activated during mouse heart development. Int J Dev Biol.

[CR26] European Commission (2020) Report from the Commission to the European Parliament and the Council. 2019 Report on the statistics on the use of animals for scientific purposes in the Member States of the European Union in 2015-2017

[CR27] FAO (2018) The State of World Fisheries and Aquaculture 2018 - Meeting the sustainable development goals. Licence: CC BY-NC-SA 3.0 IGO

[CR28] Ferron M, Settembre C, Shimazu J, Lacombe J, Kato S, Rawlings DJ, Ballabio A, Karsenty G (2013). A RANKL-PKCβ-TFEB signaling cascade is necessary for lysosomal biogenesis in osteoclasts. Genes Dev.

[CR29] Filla MS, Schwinn MK, Sheibani N, Kaufman PL, Peters DM (2009). Regulation of cross-linked actin network (CLAN) formation in human trabecular meshwork (HTM) cells by convergence of distinct β1 and β3 integrin pathways. Investig Ophthalmol Vis Sci.

[CR30] Geng X, Feng J, Liu S, Wang Y, Arias C, Liu Z (2014). Transcriptional regulation of hypoxia inducible factors alpha (HIF-α) and their inhibiting factor (FIH-1) of channel catfish (*Ictalurus punctatus*) under hypoxia. Comp Biochem Physiol B Biochem Mol Biol.

[CR31] Gracey AY, Troll JV, Somero GN (2001). Hypoxia-induced gene expression profiling in the euryoxic fish *Gillichthys mirabilis*. Proc Natl Acad Sci U S A.

[CR32] Grunow B, Noglick S, Kruse C, Gebert M (2011). Isolation of cells from Atlantic sturgeon *Acipenser oxyrinchus oxyrinchus* and optimization of culture conditions. Aquat Biol.

[CR33] Grunow B, Wenzel J, Terlau H, Langner S, Gebert M, Kruse C (2011). In vitro developed spontaneously contracting cardiomyocytes from rainbow trout as a model system for human heart research. Cell Physiol Biochem.

[CR34] Grunow B, Mohamet L, Shiels HA (2015). Generating an in vitro 3D cell culture model from zebrafish larvae for heart research. J Exp Biol.

[CR35] Güralp H, Pocherniaieva K, Blecha M, Policar T, Pšenička M, Saito T (2017). Development, and effect of water temperature on development rate, of pikeperch *Sander lucioperca* embryos. Theriogenology.

[CR36] Hamza N, Mhetli M, Kestemont P (2007). Effects of weaning age and diets on ontogeny of digestive activities and structures of pikeperch (*Sander lucioperca*) larvae. Fish Physiol Biochem.

[CR37] He C, Wang C, Li B, Xie F, Chen Y, Zuo Z (2009). Tissue-specific and embryonic expression of the retinoid X receptors in *Sebastiscus marmoratus*. Comp Biochem Physiol B Biochem Mol Biol.

[CR38] Hilge V, Steffens W (1996). Aquaculture of fry and fingerling of pike-perch (*Stizostedion lucioperca* L.) - A short review. J Appl Ichthyol.

[CR39] Hoffman LM, Garcha K, Karamboulas K, Cowan MF, Drysdale LM, Horton WA, Underhill TM (2006). BMP action in skeletogenesis involves attenuation of retinoid signaling. J Cell Biol.

[CR40] Hubenova T, Zaikov A, Katsarov E, Terziyski D (2015). Weaning of juvenile pikeperch (*Sander lucioperca* L.) from live food to artificial diet. Bulg J Agric Sci.

[CR41] Ijiri S, Kaneko H, Kobayashi T, Wang D-S, Sakai F, Paul-Prasanth B, Nakamura M, Nagahama Y (2008). Sexual Dimorphic Expression of Genes in Gonads During Early Differentiation of a Teleost Fish, the Nile Tilapia *Oreochromis niloticus*. Biol Reprod.

[CR42] Job R, Raja V, Grierson I, Currie L, O’Reilly S, Pollock N, Knight E, Clark AF (2010). Cross-linked actin networks (CLANs) are present in lamina cribrosa cells. Br J Ophthalmol.

[CR43] Kestemont P, Xueliang X, Hamza N, Maboudou J, Imorou Toko I (2007). Effect of weaning age and diet on pikeperch larviculture. Aquaculture.

[CR44] Lakra WS, Swaminathan TR, Joy KP (2011). Development, characterization, conservation and storage of fish cell lines: a review. Fish Physiol Biochem.

[CR45] Le Goff P, Le Dréan Y, Le Péron C, Le Jossic-Corcos C, Ainouche A, Michel D (2004). Intracellular trafficking of heat shock factor 2. Exp Cell Res.

[CR46] Leaver MJ, Boukouvala E, Antonopoulou E, Diez A, Favre-Krey L, Tariq Ezaz M, Bautista JM, Tocher DR, Krey G (2005). Three peroxisome proliferator-activated receptor isotypes from each of two species of marine fish. Endocrinology.

[CR47] Lee J-W, Lee J-H, Noh JK, Kim HC, Park C-J, Park J-W, Kim K-K (2014). Transcriptional Onset of Lysozyme Genes during Early Development in Olive Flounder (*Paralichthys olivaceus*). Dev Reprod.

[CR48] Leet JK, Gall HE, Sepúlveda MS (2011). A review of studies on androgen and estrogen exposure in fish early life stages: Effects on gene and hormonal control of sexual differentiation. J Appl Toxicol.

[CR49] Liu J, Plagnes-Juan E, Geurden I, Panserat S, Marandel L (2017). Exposure to an acute hypoxic stimulus during early life affects the expression of glucose metabolism-related genes at first-feeding in trout. Sci Rep.

[CR50] Löffler J, Ott A, Ahnelt H, Keckeis H (2008). Early development of the skull of *Sander lucioperca* (L.) (Teleostei: Percidae) relating to growth and mortality. J Fish Biol.

[CR51] López-Unzu MA, Durán AC, Soto-Navarrete MT, Sans-Coma V, Fernández B (2019). Differential expression of myosin heavy chain isoforms in cardiac segments of gnathostome vertebrates and its evolutionary implications. Front Zool.

[CR52] Mahley RW (1988). Apolipoprotein E : Cholesterol Transport. Science (80- ).

[CR53] Malandrakis EE, Dadali O, Golomazou E, Kavouras M, Dailianis S, Chadio S, Exadactylos A, Panagiotaki P (2016). DNA damage and differential gene expression associated with physical stress in gilthead seabream (*Sparus aurata*). Gen Comp Endocrinol.

[CR54] Martorell Ribera J, Nipkow M, Viergutz T, Brunner RM, Bochert R, Koll R, Goldammer T, Gimsa U, Rebl A (2020). Early response of salmonid head-kidney cells to stress hormones and toll-like receptor ligands. Fish Shellfish Immunol.

[CR55] Milla S, Douxfils J, Mandiki SNM, Saroglia M (2015). Chapter 28: Corticosteroids and the Stress Response in Percid Fish. Biology and Culture of Percid Fishes: Principles and Practices.

[CR56] Morimoto RI (1998). Regulation of the heat shock transcriptional response: Cross talk between a family of heat shock factors, molecular chaperones, and negative regulators. Genes Dev.

[CR57] Napolitano G, Ballabio A (2016). TFEB at a glance. J Cell Sci.

[CR58] Németh S, Horváth Z, Felföldi Z, Beliczky G, Demeter K (2013). The use of permitted ectoparasite disinfection methods on young pike-perch (*Sander lucioperca*) after transition from over-wintering lake to RAS. AACL Bioflux.

[CR59] Nguinkal JA, Brunner RM, Verleih M, Rebl A, de los Ríos-Pérez L, Schäfer N, Hadlich F, Stüeken M, Wittenburg D, Goldammer T (2019). The first highly contiguous genome assembly of pikeperch (*Sander lucioperca*), an emerging aquaculture species in Europe. Genes (Basel).

[CR60] Nipkow M, Wirthgen E, Luft P, Rebl A, Hoeflich A, Goldammer T (2018). Characterization of igf1 and igf2 genes during maraena whitefish (*Coregonus maraena*) ontogeny and the effect of temperature on embryogenesis and igf expression. Growth Hormon IGF Res.

[CR61] Ostaszewska T (2005). Developmental changes of digestive system structures in pike-perch (*Sander lucioperca* L.). Electron J Ichthyol.

[CR62] Otis JP, Zeituni EM, Thierer JH, Anderson JL, Brown AC, Boehm ED, Cerchione DM, Ceasrine AM, Avraham-Davidi I, Tempelhof H, Yaniv K, Farber SA (2015). Zebrafish as a model for apolipoprotein biology: Comprehensive expression analysis and a role for ApoA-IV in regulating food intake. DMM Dis Model Mech.

[CR63] Ott A, Löffler J, Ahnelt H, Keckeis H (2012). Early development of the postcranial skeleton of the pikeperch *Sander lucioperca* (Teleostei: Percidae) relating to developmental stages and growth. J Morphol.

[CR64] Ott A, Löffler J, Ahnelt H, Keckeis H (2012). Early development of the postcranial skeleton of the pikeperch *Sander lucioperca* (Teleostei: Percidae) relating to developmental stages and growth. J Morphol.

[CR65] Padmini E, Usha Rani M (2009). Seasonal influence on heat shock protein 90α and heat shock factor 1 expression during oxidative stress in fish hepatocytes from polluted estuary. J Exp Mar Biol Ecol.

[CR66] Pavlidis M, Karantzali E, Fanouraki E, Barsakis C, Kollias S, Papandroulakis N (2011). Onset of the primary stress in European sea bass *Dicentrarhus labrax*, as indicated by whole body cortisol in relation to glucocorticoid receptor during early development. Aquaculture.

[CR67] Pelster B, Egg M (2018). Hypoxia-inducible transcription factors in fish: expression, function and interconnection with the circadian clock. J Exp Biol.

[CR68] Policar T, Blecha M, Křišťan J, Mráz J, Velíšek J, Stará A, Stejskal V, Malinovskyi O, Svačina P, Samarin AM (2016). Comparison of production efficiency and quality of differently cultured pikeperch (*Sander lucioperca* L.) juveniles as a valuable product for ongrowing culture. Aquac Int.

[CR69] Poupard G, André M, Durliat M, Ballagny C, Boeuf G, Babin PJ (2000). Apolipoprotein E gene expression correlates with endogenous lipid nutrition and yolk syncytial layer lipoprotein synthesis during fish development. Cell Tissue Res.

[CR70] Raben N, Puertollano R (2016). TFEB and TFE3: Linking Lysosomes to Cellular Adaptation to Stress. Annu Rev Cell Dev Biol.

[CR71] Roberts RJ, Agius C, Saliba C, Bossier P, Sung YY (2010). Heat shock proteins (chaperones) in fish and shellfish and their potential role in relation to fish health: A review. J Fish Dis.

[CR72] Rojas DA, Perez-Munizaga DA, Centanin L, Antonelli M, Wappner P, Allende ML, Reyes AE (2007). Cloning of hif-1α and hif-2α and mRNA expression pattern during development in zebrafish. Gene Expr Patterns.

[CR73] Rytkönen KT, Vuori KAM, Primmer CR, Nikinmaa M (2007). Comparison of hypoxia-inducible factor-1 alpha in hypoxia-sensitive and hypoxia-tolerant fish species. Comp Biochem Physiol D Genomics Proteomics.

[CR74] Saurabh S, Sahoo PK (2008). Lysozyme: An important defence molecule of fish innate immune system. Aquac Res.

[CR75] Schaefer FJ, Flues S, Meyer S, Peck MA (2017). Inter- and intra-individual variability in growth and food consumption in pikeperch, *Sander lucioperca* L., larvae revealed by individual rearing. Aquac Res.

[CR76] Schaefer FJ, Overton JL, Krüger A, Kloas W, Wuertz S (2018). Influence of batch-specific biochemical egg characteristics on embryogenesis and hatching success in farmed pikeperch. Animal.

[CR77] Schlueter PJ, Sang X, Duan C, Wood AW (2007). Insulin-like growth factor receptor 1b is required for zebrafish primordial germ cell migration and survival. Dev Biol.

[CR78] Schlumberger O, Proteau JP (1996). Reproduction of pike-perch (*Stizostedion lucioperca*) in captivity. J Appl Ichthyol.

[CR79] Settembre C, Ballabio A (2011). TFEB regulates autophagy: An integrated coordination of cellular degradation and recycling processes. Autophagy.

[CR80] Settembre C, De Cegli R, Mansueto G, Saha PK, Vetrini F, Visvikis O, Huynh T, Carissimo A, Palmer D, Jürgen Klisch T, Wollenberg AC, Di Bernardo D, Chan L, Irazoqui JE, Ballabio A (2013). TFEB controls cellular lipid metabolism through a starvation-induced autoregulatory loop. Nat Cell Biol.

[CR81] Shen RJ, Jiang XY, Pu JW, Zou SM (2010). HIF-1α and -2α genes in a hypoxia-sensitive teleost species *Megalobrama amblycephala*: cDNA cloning, expression and different responses to hypoxia. Comp Biochem Physiol B Biochem Mol Biol.

[CR82] Steenfeldt S (2015). Chapter 10: Culture Methods of Pikeperch Early Life Stages. Biology and Culture of Percid Fishes: Principles and Practices.

[CR83] Streelman JT, Webb JF, Albertson RC, Kocher TD (2003). The cusp of evolution and development: A model of cichlid tooth shape diversity. Evol Dev.

[CR84] Swirplies F, Wuertz S, Baßmann B, Orban A, Schäfer N, Brunner RM, Hadlich F, Goldammer T, Rebl A (2019). Identification of molecular stress indicators in pikeperch *Sander lucioperca* correlating with rising water temperatures. Aquaculture.

[CR85] Szkudlarek M, Zakȩś Z (2007). Effect of stocking density on survival and growth performance of pikeperch, *Sander lucioperca* (L.), larvae under controlled conditions. Aquac Int.

[CR86] Tan T, Yu RMK, Wu RSS, Kong RYC (2017). Overexpression and Knockdown of Hypoxia-Inducible Factor 1 Disrupt the Expression of Steroidogenic Enzyme Genes and Early Embryonic Development in Zebrafish. Gene Regul Syst Bio.

[CR87] Tang JLY, Guo Y, Stockdale WT, Rana K, Killen AC, Mommersteeg MTM, Yamamoto Y (2018). The developmental origin of heart size and shape differences in *Astyanax mexicanus* populations. Dev Biol.

[CR88] Tiller GR, Garsin DA (2014). Of Worms and Men: HLH-30 and TFEB Regulate Tolerance to Infection. Immunity.

[CR89] Tsalafouta A, Sarropoulou E, Papandroulakis N, Pavlidis M (2018). Characterization and Expression Dynamics of Key Genes Involved in the Gilthead Sea Bream (*Sparus aurata*) Cortisol Stress Response during Early Ontogeny. Mar Biotechnol.

[CR90] Tse WKF (2014). The role of osmotic stress transcription factor 1 in fishes. Front Zool.

[CR91] Vikstrom KL, Seiler SH, Sohn RL, Strauss M, Weiss A, Welikson RE, Leinwand LA (1997). The vertebrate myosin heavy chain: Genetics and assembly properties. Cell Struct Funct.

[CR92] Vuori KAM, Soitamo A, Vuorinen PJ, Nikinmaa M (2004). Baltic salmon (Salmo salar) yolk-sac fry mortality is associated with disturbances in the function of hypoxia-inducible transcription factor (HIF-1α) and consecutive gene expression. Aquat Toxicol.

[CR93] Wang Y, Li C, Pan C, Liu E, Zhao X, Ling Q (2019) Alterations to transcriptomic profile, histopathology, and oxidative stress in liver of pikeperch (*Sander lucioperca*) under heat stress. Fish Shellfish Immunol 95:659–651. 10.1016/J.FSI.2019.11.014 10.1016/j.fsi.2019.11.014 31706008

[CR94] Whitehouse LM, Manzon RG (2019). Hypoxia alters the expression of hif-1a mRNA and downstream HIF-1 response genes in embryonic and larval lake whitefish (*Coregonus clupeaformis*). Comp Biochem Physiol -Part A Mol Integr Physiol.

[CR95] Wiegand MD (1996). Composition, accumulation and utilization of yolk lipids in teleost fish. Rev Fish Biol Fish.

[CR96] Wilensky C, Bowser R (2005). Growth characteristics of the WF2 cell cultures. J World Aquacult Soc.

[CR97] Woltering DM (1984). The growth response in fish chronic and early life stage toxicity tests: A critical review. Aquat Toxicol.

[CR98] Wyss M, Kaddurah-Daouk R (2000). Creatine and creatinine metabolism. Physiol Rev.

[CR99] Yokoi H, Kobayashi T, Tanaka M, Nagahama Y, Wakamatsu Y, Takeda H, Araki K, Morohashi KI, Ozato K (2002). Sox9 in a teleost fish, medaka (*Oryzias latipes*): Evidence for diversified function of Sox9 in gonad differentiation. Mol Reprod Dev.

[CR100] Zapata A, Diez B, Cejalvo T, de Gutiérrez Frías C, Cortés A (2006). Ontogeny of the immune system of fish. Fish Shellfish Immunol.

[CR101] Żarski D, Ben Ammar I, Bernáth G, Baekelandt S, Bokor Z, Palińska-Żarska K, Fontaine P, Horváth Á, Kestemont P, Mandiki SNM (2020). Repeated hormonal induction of spermiation affects the stress but not the immune response in pikeperch (*Sander lucioperca*). Fish Shellfish Immunol.

[CR102] Zou J, Secombes CJ (2016) The function of fish cytokines. Biol (Basel) 5. 10.3390/biology5020023 10.3390/biology5020023 PMC4929537 27231948

